# Non-Cell-Autonomous Mechanisms in Radial Projection Neuron Migration in the Developing Cerebral Cortex

**DOI:** 10.3389/fcell.2020.574382

**Published:** 2020-09-25

**Authors:** Andi H. Hansen, Simon Hippenmeyer

**Affiliations:** Institute of Science and Technology Austria, Klosterneuburg, Austria

**Keywords:** cerebral cortex, radial projection neuron migration, non-cell-autonomous mechanisms, neurodevelopmental migration disorders, single cell analysis

## Abstract

Concerted radial migration of newly born cortical projection neurons, from their birthplace to their final target lamina, is a key step in the assembly of the cerebral cortex. The cellular and molecular mechanisms regulating the specific sequential steps of radial neuronal migration *in vivo* are however still unclear, let alone the effects and interactions with the extracellular environment. In any *in vivo* context, cells will always be exposed to a complex extracellular environment consisting of (1) secreted factors acting as potential signaling cues, (2) the extracellular matrix, and (3) other cells providing cell–cell interaction through receptors and/or direct physical stimuli. Most studies so far have described and focused mainly on intrinsic cell-autonomous gene functions in neuronal migration but there is accumulating evidence that non-cell-autonomous-, local-, systemic-, and/or whole tissue-wide effects substantially contribute to the regulation of radial neuronal migration. These non-cell-autonomous effects may differentially affect cortical neuron migration in distinct cellular environments. However, the cellular and molecular natures of such non-cell-autonomous mechanisms are mostly unknown. Furthermore, physical forces due to collective migration and/or community effects (i.e., interactions with surrounding cells) may play important roles in neocortical projection neuron migration. In this concise review, we first outline distinct models of non-cell-autonomous interactions of cortical projection neurons along their radial migration trajectory during development. We then summarize experimental assays and platforms that can be utilized to visualize and potentially probe non-cell-autonomous mechanisms. Lastly, we define key questions to address in the future.

## Introduction

**FIGURE 1:**
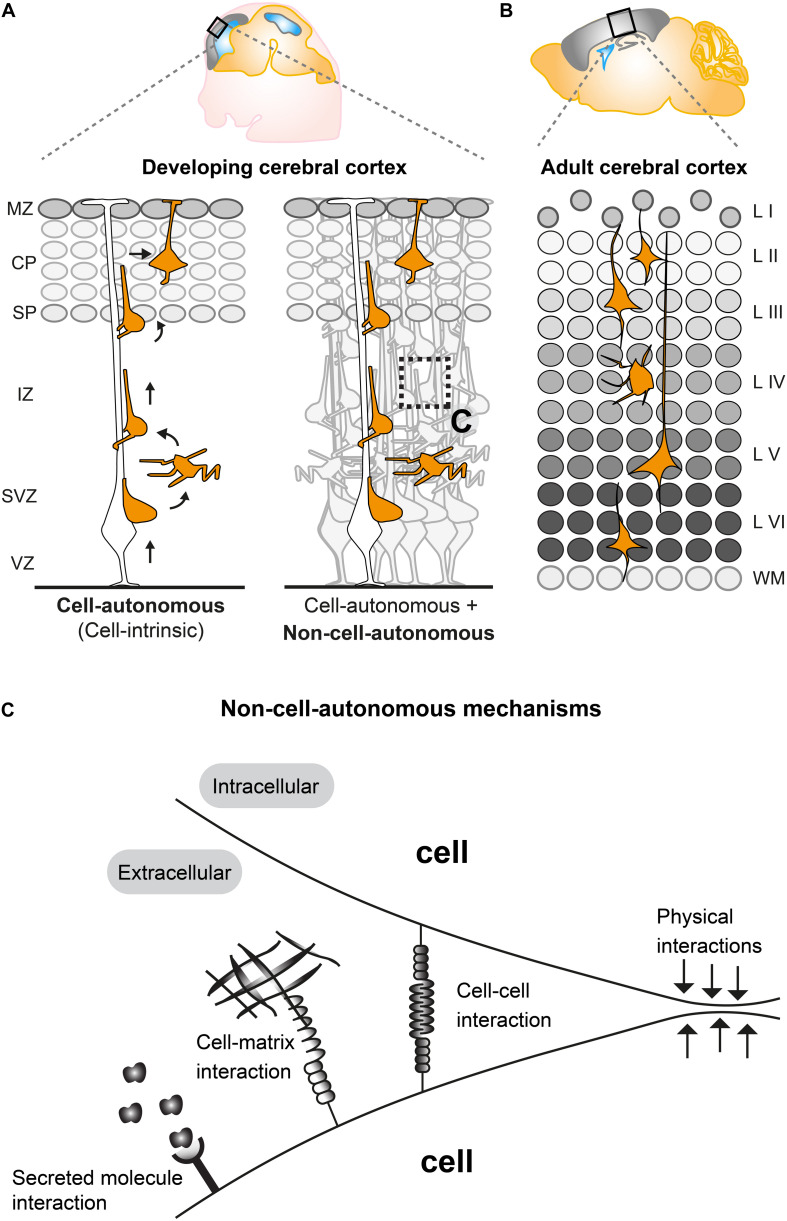
Non-cell-autonomous mechanisms in radial projection neuron migration. **(A)** Migrating cortical projection neurons go through several steps and phases during their journey from their birthplace in the ventricular/subventricular zone (VZ/SVZ) to their final position in the CP. In the left panel, an isolated radially migrating projection neuron is shown to illustrate intrinsic cell-autonomous mechanisms controlling radial migration. The right panel illustrates that radially migrating projection neurons, which are embedded in an environment consisting of many other cells, are potentially influenced (in addition to cell intrinsic cues) through non-cell-autonomous mechanisms (See panel **C**). **(B)** The six layered (I–VI) structure of the adult mouse cerebral cortex. The layers are assembled in an inside out fashion where layers V, VI are the earliest generated and layers II–IV the latest generated cortical projection neurons. **(C)** Possible non-cell-autonomous cellular and molecular interactions during radial projection neuron migration. In any *in vivo* context, cells will always be exposed to a complex extracellular environment consisting of secreted factors acting as potential signaling cues, the extracellular matrix and other cells providing cell–cell interaction through receptors and/or direct physical stimuli. VZ, ventricular zone; SVZ, subventricular zone; IZ, intermediate zone; SP, subplate; CP, cortical plate; WM, white matter; L I-VI, layers 1–6.

The mammalian neocortex is built by distinct classes of neurons and glial cells which are organized into six stratified layers. Here we focus on projection neurons, the major neuronal population in the cortex. Projection neurons emerge from radial glial cells (RGCs) in the ventricular zone (VZ), intermediate progenitor cells (IPCs), and outer radial glial cells (oRGs, aka basal radial glia, bRGs) which divide in the subventricular zone (SVZ) (Ayala et al., 2007; Hansen et al., 2010; Wang et al., 2011; Borrell and Götz, 2014). Nascent projection neurons migrate from their place of origin in the VZ/SVZ to their final target position, a process which is highly regulated (Ayala et al., 2007; Valiente and Marín, 2010; Evsyukova et al., 2013). Concerted migration of sequentially generated projections neurons results in a neocortex which is structured into six distinct layers (I–VI), each with different cellular composition and arranged in an inside-out fashion (McConnell, 1995; Lodato and Arlotta, 2015) (Figures 1A,B). In order to establish the correct cortical layering during development, projection neurons exhibit radial migration from the VZ/SVZ to the cortical plate (CP). Around embryonic day 11 (E11), post-mitotic neurons migrate mainly by pulling up the soma in the upright direction by using a basal process that is firmly attached to the pial surface. This migration mode is termed somal translocation (Nadarajah et al., 2001). The first cohort of migrating neurons form the preplate (PP), a structure which only exists transiently (Allendoerfer and Shatz, 1994; Nadarajah et al., 2001). At around E12, consecutive waves of neurons migrate toward the pial surface and establish the CP by splitting the PP into the two distinct structures: the deeper located subplate (SP) and the superficially positioned marginal zone (MZ) (layer I) (Ayala et al., 2007) (Figure 1A). The subsequent populations of migrating neurons establish the ‘first’ layer of projection neurons (i.e., layer VI) in the CP which progressively expands in the vertical direction in an inside-out manner (Figure 1). In other words, earlier generated neurons settle in the deeper layers (layers V, VI) whereas later generated neurons migrate through the deep positioned neurons creating more superficial layers (II, IV) (Angevine and Sidman, 1961; McConnell, 1995; Valiente and Marín, 2010).

Studies applying histological and time-lapse imaging techniques have shed some light on the dynamics of the radial migration process and described distinct sequential steps of projection neuron migration (Figure 1A) (Nadarajah et al., 2003; Tabata and Nakajima, 2003; Noctor et al., 2004). Newly-born neurons delaminate from the VZ and move toward the SVZ where they accumulate in the lower part and acquire a multipolar shape, characterized by multiple processes pointing in different directions (Tabata et al., 2009). In the SVZ, multipolar neurons move tangentially, toward the pia or toward the VZ (Tabata and Nakajima, 2003; Noctor et al., 2004). Multipolar neurons can remain up to 24 h in the multipolar state in the SVZ. Next, within the SVZ and the lower part of the intermediate zone (IZ) multipolar neurons switch back to a bipolar state with a ventricle-oriented process that eventually develops into the axon. The pial oriented leading process is established by reorienting the Golgi and the centrosome toward the pial surface (Hatanaka et al., 2004; Yanagida et al., 2012). Upon multi-to-bipolar transition, neurons attach to the radial glial fiber in the upper part of the IZ and move along RGCs in a migration mode termed locomotion, while trailing the axon behind and rapidly extending and retracting their leading neurite before reaching the SP (Hatanaka et al., 2004; Noctor et al., 2004). Neurons then cross the SP and enter the CP still migrating along the RGCs until they reach the marginal zone (MZ). Just beneath the MZ neurons stop locomoting and detach from the radial glia fiber to perform terminal somal translocation and settle in their target position where they eventually assemble into microcircuits (Rakic, 1972; Nadarajah et al., 2001; Noctor et al., 2004; Hatanaka et al., 2016). All sequential steps of projection neuron migration are critical and disruption at any stage (e.g., due to genetic mutations in genes encoding core migration machinery) can lead to severe cortical malformations (Gleeson and Walsh, 2000; Guerrini and Parrini, 2010). Therefore each step of projection neuron migration must be tightly regulated. Many genes have been identified as causative factors for cortical malformations (Heng et al., 2010; Valiente and Marín, 2010; Evsyukova et al., 2013) and several of the key molecules involved in neuronal migration, e.g., LIS1, DCX, and REELIN have been investigated in detail by molecular genetics (Kawauchi, 2015). Recently, approaches involving *in vivo* electroporation and time-lapse imaging of brain slice cultures have shed light on crucial roles for the dynamic regulation of the cytoskeleton, extracellular cues and cell adhesion during neuronal migration (Noctor et al., 2004; Schaar and McConnell, 2005; Simo et al., 2010; Franco et al., 2011; Jossin and Cooper, 2011; Sekine et al., 2012). An emerging picture is arising with distinct molecular programs regulating neuronal migration through the different compartments VZ/SVZ, IZ, and CP (Kwan et al., 2012; Greig et al., 2013; Hippenmeyer, 2014; Hansen et al., 2017; Jossin, 2020). However, the precise regulatory mechanisms which coordinate each and every specific step of radial migration are still largely unknown, let alone the effects and interactions with the extracellular environment. Most studies so far have described and focused mainly on intrinsic cell-autonomous gene functions (Figure 1A) in neuronal migration (reviewed in Heng et al., 2010; Valiente and Marín, 2010; Evsyukova et al., 2013) but there is accumulating evidence that non-cell- autonomous-, local-, systemic- and/or whole tissue-wide effects (Figures 1A,C) substantially contribute to the regulation of radial neuronal migration (Hammond et al., 2001; Yang et al., 2002; Sanada et al., 2004; Youn et al., 2009; Hippenmeyer et al., 2010; Franco et al., 2011; Hippenmeyer, 2014; van den Berghe et al., 2014; Gorelik et al., 2017; Nakagawa et al., 2019).

## Nature of Non-Cell-Autonomous Mechanisms in Radial Projection Neuron Migration

In any *in vivo* context, cells will always be exposed to a complex extracellular environment consisting of (1) secreted factors acting as potential signaling cues, (2) the extracellular matrix, and (3) other cells providing cell–cell interaction through receptors and/or direct physical stimuli (Figure 1C). Therefore, most genes controlling radial neuronal migration can potentially, besides cell-autonomous functions, also act through non-cell-autonomous mechanisms. As such, non-cell-autonomous regulatory cues could involve molecular, cellular, or physical components (Figure 1C). Hence, the distinction between cell-autonomous gene function and non-cell-autonomous mechanisms is important to be able to define the different facets of a gene function *in vivo* and thus intact tissue context. Below we will describe recent studies and findings which have started to describe and characterize non-cell-autonomous effects and mechanisms in projection neuron migration.

### Secreted Molecules and the Extracellular Matrix

One of the most apparent non-cell-autonomous interactions includes secreted molecules produced in one cell and eliciting a response in another cell. In addition, interactions with the extracellular matrix are bound to happen for any cell and can occur in various ways. The extracellular matrix provides both structural organization of the cerebral cortex as well as the control of individual neurons. Neuronal migration and lamination is organized by extracellular matrix glycoproteins such as, e.g., laminins, tenascins, proteoglycans, and Reelin (Barros et al., 2011). The specific type of interaction of neurons with secreted molecules and the extracellular matrix and their role in radial neuronal migration have been reviewed recently in detail elsewhere (Franco and Müller, 2011; Maeda, 2015; Long and Huttner, 2019). Here we will briefly elaborate upon a few secreted molecules, mainly Reelin, which play roles in neuronal migration and brain development in general. The Reelin/Dab1 signaling cascade represents one of the best characterized signaling pathways in the developing brain. Reelin is a secreted protein mainly expressed by Cajal-Retzius cells in the MZ of the cortex (Ogawa et al., 1995) and acts via DAB1 in the control of radial projection neuron migration (Rice et al., 1998; Honda et al., 2011). The originally isolated *reeler* mouse mutant and *Dab1* KO mice show a severe disorganization of cortical projection neurons resembling a neocortex layering which is more or less inverted (Caviness and Sidman, 1973). Reelin has been hypothesized to inherit a number of distinct signaling modalities and functions in cortical neuronal migration (Honda et al., 2011; D’Arcangelo, 2014) but the precise role in the local microenvironment of migrating projection neurons is not clear (Jossin, 2020). Yet, Reelin is mainly secreted from the CR-cells in the MZ and processed Reelin fragments has been shown to diffuse from the MZ into the CP and IZ of the developing cortex (Jossin et al., 2007; D’Arcangelo, 2014; Koie et al., 2014). Interestingly, when Reelin is ectopically expressed and secreted by migrating neurons in the IZ, it leads to aggregation of neurons near this ectopic Reelin-rich region resembling the structure of the MZ (Kubo et al., 2010). Furthermore, sequential labeling of migrating neurons revealed that the late-born neurons can still pass by the early-born neurons during the formation of an ectopic Reelin rich aggregate (Kubo et al., 2010). These results indicate that Reelin may have distinct roles in long range versus local signaling. Moreover, a recent study investigating a FMCD-causing (Focal malformations of cortical development) mutation revealed that over activation of AKT3 in a fraction of migrating neurons would lead to misexpression of Reelin in these cells and thereby affect the migration of wild-type neighboring cells in a non-cell-autonomous manner (Baek et al., 2015). Moreover, RNA-seq expression profiling was employed to further investigate the non-cell-autonomous migration defect which could be due to direct physical blockade of the wild-type cells or have a more specific signaling mechanism. The gene ontology enrichment of the 835 significantly deregulated genes identified four main categories for neuronal development, migration, signaling and homeostasis and cell cycle regulation. This suggests that the non-cell-autonomous defect might underlie a more complicated mechanism than just a simple blockade of neurons (Baek et al., 2015). Clearly, the above studies show that global or local expression of a secreted molecule can cause distinct phenotypes, and demonstrating significant non-cell-autonomous impact on projection neuron migration.

Reelin signaling in the control of radial projection neuron migration acts via the intracellular adaptor protein DAB1 (Rice et al., 1998; Honda et al., 2011). Studies applying genetically engineered chimeric mice have suggested that environmental conditions play a role in proper neuronal positioning, and proposed a non-cell-autonomous effect and/or element of *Dab1* function (Hammond et al., 2001; Yang et al., 2002). By using conditional-KO (cKO) mice, in which *Dab1* is specifically deleted after preplate splitting and only in late-born neurons, it was observed that wild-type early born neurons were positioned in the outer layers instead of their usual position in the inner cortical layers. This would suggest that early-born neurons are being “passively” displaced into a deeper position by later-born neurons (Franco et al., 2011). Taken together, the pleiotropy of Reelin-Dab1 loss of function phenotypes could be significantly affected by non-cell-autonomous effects elicited by environmental factors and/or community effects in addition to the cell-autonomous function of Reelin signaling on migrating neurons.

Fibroblast growth factors (FGFs) is a family of secreted molecules and their receptors (FGFRs) were recently shown to play an important role in radial projection neuron migration (Ford-Perriss et al., 2001; Ornitz and Itoh, 2015; Szczurkowska et al., 2018; Kon et al., 2019). A recent study implicated FGFRs in the regulation of the migration orientation of multipolar neurons and the multipolar-to-bipolar transition. It was shown that FGFRs are activated by N-Cadherin when binding in *cis* on the same cell which prevents degradation and results in accumulation of FGFR which stimulate prolonged activation of extracellular signal-regulated kinase (Erk1/2) required for multipolar migration (Kon et al., 2019). In another study, NEGR1, another cell adhesion molecule, was shown to interact with FGFR2 thereby regulating neuronal migration and spine density (Szczurkowska et al., 2018). This study showed that NEGR1 physically interacts with FGFR2 and prevents it from being transported for lysosomal degradation. This accumulation of FGFR2 results in the maintenance of downstream ERK and AKT signaling. These two above studies have shown that FGFR receptors are important in neuronal migration, however the exact response mechanism of secreted FGF ligands is currently unknown. Since a large number of FGFs are expressed in the developing cortex and FGFRs are also activated by heparan sulfate proteoglycans, it is challenging to investigate which and how a specific FGF is involved in neuronal migration (Ford-Perriss et al., 2001; Ornitz and Itoh, 2015). The fact that FGFRs physically interact with different cell adhesion molecules, but act on similar downstream signaling pathways important for neuronal migration, indicates an important general role of FGFR signaling.

Recent findings suggest that alteration of individual neurons might also affect the entire cellular community. As such, a screen identified several potential non-cell autonomous regulators of radial neuronal migration and described autotaxin (ATX) to affect the localization and adhesion of neuronal progenitors in a cell autonomous and non-cell autonomous manner (Greenman et al., 2015). In a follow-up study, *Serping1*, a candidate gene identified in the above screen, was found to be expressed and secreted by neurons during brain development and to both affect radial neuronal migration in a cell-autonomous and non-cell-autonomous way (Gorelik et al., 2017). Besides affecting the positioning of the neurons, loss of *Serping1* gene function would also affect the cellular morphology of the neighboring neurons since knockdown neurons exhibited long leading processes which were also observed in the adjacent non-manipulated neurons (Gorelik et al., 2017).

### Cell–Cell Interactions Among Migrating Cortical Projection Neurons

It has been observed that migrating neurons can have a positive and negative influence on each other depending on their genetic constitution and the environment. However, the nature of potential positive and/or negative non-cell-autonomous effects and how they affect the migration of mutant and wild-type cortical projection neurons is currently unclear (Hippenmeyer, 2014). Cell–cell interactions during collective cell migration, in a variety of cell types, have indeed been observed previously. Interactions mainly occur when two or more cells that retain their cell–cell contacts move together while coordinating their actin dynamics and intracellular signaling (Friedl and Gilmour, 2009; Tada and Heisenberg, 2012; Londono et al., 2014). Studies looking at collective migration, e.g., in neural crest cells has provided information for the understanding of balanced interaction of cell adhesion and cell signaling between collectively migrating cells. Balancing adhesion and repulsion is one major factor mediating both individual cell and collective migratory coordination (Shellard and Mayor, 2020). Therefore, collective decision making and organization of cells is crucial for the generation of complex tissue and could also apply for the assembly of the cerebral cortex which relies on the migration of neurons. An example of such an collective effect could be physical properties where mutant (which may be less agile) neurons either “piggyback” on adjacent normally migrating neurons or get passively pushed or pulled by a migrating cellular population. Collective influences could also have a negative effect if most or all neurons are mutant and less dynamic, thereby leading to improper migration. Another effect of surrounding neurons could be through signaling, to stimulate or tune down the intrinsic migratory machinery of deficient neurons. This would suggest a mechanism whereby active signaling is utilized through transmembrane receptors and/or extracellular matrix components. Indeed such mechanisms have been described in various cell types where mutant cells negatively affect migration by direct contact inhibition (Huttenlocher et al., 1998; Becker et al., 2013). Upon ectopic expression of cell adhesion molecules, such as N-cadherin, Integrin, Focal adhesion kinase and the focal-adhesion adaptor protein Paxillin in cell culture, direct cell–cell contact inhibited migration. Interestingly, when mutant cells were surrounded by wild-type cells no such effect was seen. Nevertheless, when mutant cells were in direct contact with other mutant cells then the migratory process was inhibited (Huttenlocher et al., 1998; Becker et al., 2013). Although this effect was shown *in vitro* it could also apply to migrating projection neurons *in vivo*. However, in the case of N-cadherin, the cause of inhibited migration could be due to intracellular trafficking and abundance of N-Cadherin rather than expression itself. A study has shown that Rab5-dependent endocytotic-, and a Rab11-dependent recycling pathway regulate N-cadherin trafficking, thereby mediating adhesion between a migrating projection neuron and the radial glial fiber (Kawauchi et al., 2010).

*In vivo* studies have recently shown that mutant *Ndel1* MADM (mosaic analysis with double markers)-labeled neurons, surrounded by a normal environment, exhibit different migration phenotypes when compared to mutant projection neurons in whole cortex knockout (Youn et al., 2009; Hippenmeyer et al., 2010; Hippenmeyer, 2014). *Ndel1* mutant neurons were incapable of moving in mice with a complete loss of *Ndel1* in the whole cortex, whereas *Ndel1* mutant neurons could migrate through the VZ/SVZ/IZ in a mosaic environment containing wild-type, heterozygous and mutant neurons (Youn et al., 2009; Hippenmeyer et al., 2010). Thus, the comparison of mutant *Ndel1* neurons in mutant versus normal environment clearly suggests a major influence of tissue-wide and/or community effects on radial projection neuron migration. However, the molecular and cellular mechanisms that differentially affect mutant *Ndel1* projection neurons in distinct environments remain unknown. Interestingly, differential gene expression analysis of brains from wild-type mice and full knock out mouse models for *Ndel1* (and *Lis1*, and *Ywhae* acting in the same signaling pathway) have revealed that cell adhesion, and cytoskeleton organization pathways are commonly altered in these mutants (Pramparo et al., 2011). Since cell adhesion is one of the commonly identified deregulated pathways, it would be obvious to speculate that the non-cell-autonomous response could be emerging from cell–cell or cell–matrix interactions and in the end cause the developmental phenotype observed in, e.g., *Ndel1* knockout mice.

P35 is the main activator of CDK5, a serine/threonine kinase mainly expressed in the brain (Su and Tsai, 2011; Kawauchi, 2014). In a study investigating *p35*, it was found that when rescuing *p35* in a subset of neurons in an otherwise *p35*-deficient environment, rescued neurons would migrate ‘normally’ like wild-type neurons, indicating a prominent cell-autonomous gene function of *p35* (Gupta et al., 2003). However, in a follow-up study using *p35* chimeras (creating a mix of wild-type and *p35* deficient neurons), a partial non-cell-autonomous rescue of *p35* mutant neurons was seen. Interestingly, within the *p35* chimeras it was observed that mutant cells were always present in a higher proportion compared to wild-type cells. These data indicate a certain degree of disadvantage of the wild-type neurons within the mutant cortical landscape, which could be due to non-cell-autonomous effects (Hammond, 2004). While p35/Cdk5 signaling may significantly influence how neurons interact with one another the nature of these interactions are currently unclear. These interactions however likely involve cell–cell adhesion and/or other community effects (Kwon et al., 2000; Hammond, 2004; Kawauchi, 2012, 2014). Interestingly, the Reelin-DAB1 pathway (see above) has also been shown to control cell-adhesion during neuronal migration (Sekine et al., 2014). Thus a common component of the underlying mechanisms inherent to non-cell-autonomous effects, and as observed in *p35* and *Dab1* mutant, may be acting through similar cell-adhesion signaling modules.

### Heterogeneous Cell–Cell Interactions of Migrating Cortical Projection Neurons

The developing brain consists of a heterogeneous mix of different cell types. Therefore, cell–cell interaction between distinct cell types, e.g., a radial glial cell and a migrating neuron, is one such example. Most radially migrating neurons are dependent on the radial glial fiber on which they locomote to move toward the pial surface and surpass earlier born neurons (Rakic, 1972; Nadarajah et al., 2001; Kriegstein and Noctor, 2004). Hence, the migrating neurons are dependent on a proper RGC fiber grid to be able to migrate properly. Indeed, disruption of the proper organization of the RGC fiber grid leads to non-cell-autonomous migration phenotypes because the main substrate of migrating neurons is perturbed (Belvindrah et al., 2007; Cappello et al., 2012; Nakagawa et al., 2019). Such findings initially emerged in a study investigating beta1 integrins in neuronal development. In a KO mouse model which lacks beta1 integrin in the entire central nervous system, consequently in both radial glia cells and neurons, the formation of cortical layers were affected due to perturbations in the radial glial end feet contacting the marginal zone (Graus-Porta et al., 2001). Moreover, the morphology of the apical dendrites of the pyramidal neurons was also perturbed. However, when ablating beta1 integrin specifically in neurons that migrate along radial glial fibers, and not in the radial glia cells themselves, no neurodevelopmental defect was observed (Belvindrah et al., 2007). These findings clearly showed that when one indispensable cell type (in this case the radial glial cell) was impaired, it indirectly affected the migrating neurons and resulted in disrupted layering of the cortex due to non-cell-autonomous effects (Belvindrah et al., 2007). Furthermore, investigation of the interaction of Cajal Retzius (CR) cells and migrating neurons has shown that perturbation of *Nectin1* function in CR cells alone would affect the interaction of CR cells and the leading processes of migrating neurons (Gil-Sanz et al., 2013). This altered interaction non-cell-autonomously disturbed radial glial cell-independent somal translocation of radially migrating neurons in the cortical plate (Gil-Sanz et al., 2013).

A recent study investigating *Memo1* showed that cKO in neurons and glia would cause excessive branching of the basal processes of the RGCs resulting in altered tiling of the RGC scaffolding grid and aberrant lamination of neurons (Nakagawa et al., 2019). However, deletion of *Memo1* only in post-mitotic neurons, and not RGCs, did not affect neuronal migration. Therefore, the altered tiling of the RGCs non-cell-autonomously perturbed neuronal migration and thereby caused abnormal lamination of the cortex (Nakagawa et al., 2019).

In *Flrt1/3* double-knockout mice, which develop macroscopic cortical sulci, it was found that the lack of *Flrt1/3* resulted in reduced intercellular adhesion which lead to a mild acceleration of radially migrating neurons and enhanced clustering of neurons along the tangential axis (del Toro et al., 2017). The clustering of neurons was hypothesized to result from repulsive interactions with neighboring neurons and radial glial cells suggesting a non-cell-autonomous effect of the *Flrt1/3* ablation on radial neuronal migration (Seiradake et al., 2014; del Toro et al., 2017). In a subsequent study it was shown that Teneurins, Latrophillins and FLRTs interact and direct radial neuronal migration by slowing down migration by possible coincidence contact repulsion between the neurons and the radial glia cells (del Toro et al., 2020).

Taken altogether, the above observations suggest that neuronal migration and proper lamination of the developing neocortex are significantly affected by non-cell-autonomous components. However, the precise underlying cellular and molecular mechanisms of non-cell-autonomous effects on radial neuronal migration have yet to be explored by rigorous qualitative and quantitative means. The lack of information on non-cell-autonomous effects is mainly due to the limitation of experimental assays that allow for investigation of such events *in vivo* and with single cell resolution. To this end, in the below section we illustrate contemporary experimental paradigms that have the potential to systematically analyze non-cell-autonomous mechanisms in radial migration of cortical projection neurons.

## Cellular Assays to Analyze and Genetically Dissect Non-Cell-Autonomous Mechanisms in Cortical Projection Neuron Migration *in vivo*

In this section we will specifically elaborate on the experimental paradigms which can be utilized to dissect non-cell-autonomous mechanisms in cortical projection neuron migration.

### Chimeras

**FIGURE 2:**
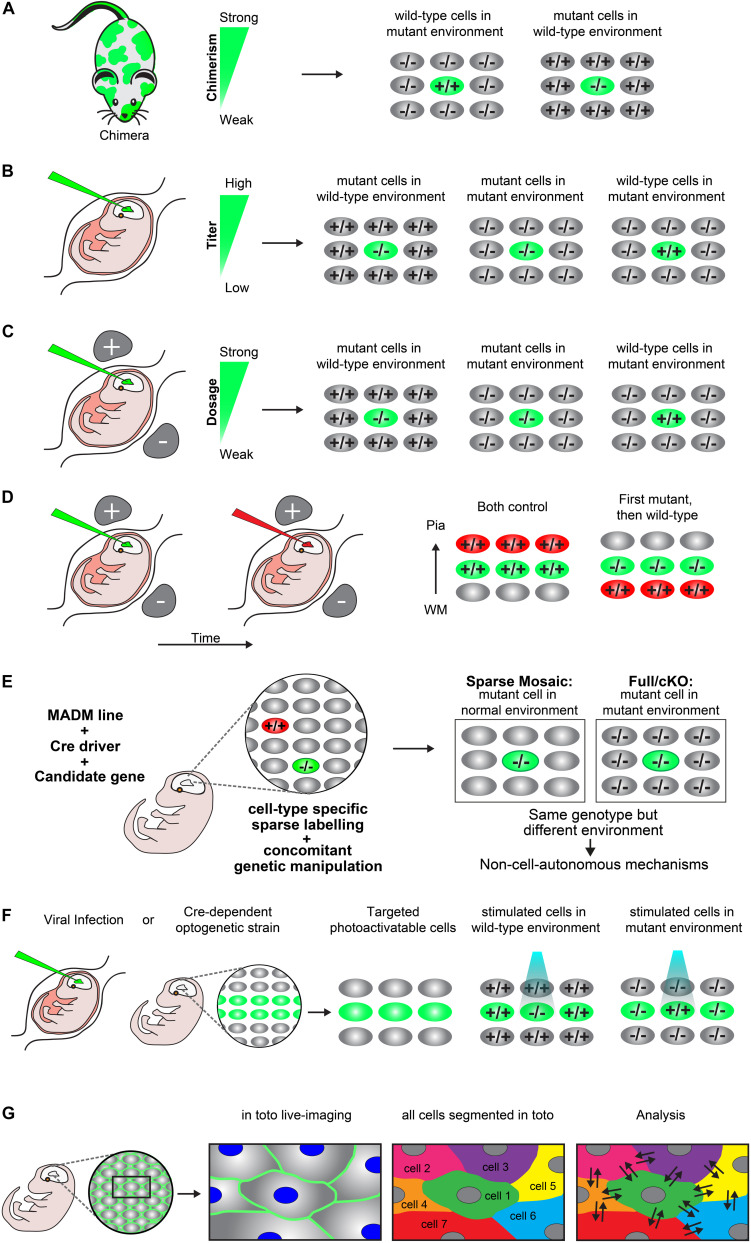
Experimental paradigms to genetically dissect non-cell-autonomous mechanisms in radial cortical neuron migration. (**A**) Chimeras. A chimera is an animal that has two or more populations of genetically distinct cells. Depending on the degree of chimerism (i.e., ratio of wild-type versus mutant cells), such assay offers one way to distinguish between cell-autonomous gene function and non-cell-autonomous mechanisms *in vivo*. Any phenotypic difference seen between the neurons of the same genotype, but present in distinct genotypic environments indicate non-cell-autonomous effects. **(B)** Retroviral infection. Retroviral infection allows to sparsely target developing neurons by either expression of the reporter only (e.g., in a wild-type or mutant environment) or using a viral vector that encodesa wild-type or mutant version of the gene of interest in combination with a reporter. This facilitates the inactivation or rescue of the gene of interest in either wild-type or mutant environments, allowing for the distinction of cell-autonomous gene function and non-cell-autonomous effects. Appropriately diluted retrovirus encoding the reporter and gene of interest allows for the discrimination of individual neurons and one can adjust the viral titer to generate more or less sparsely targeted neuronal populations. **(C)** *In utero* electroporation. Timed *in utero* electroporation for inactivation of a gene allows the sparse targeting of nascent migrating neurons in an otherwise wild-type environment. The inactivation of a specific gene can either be achieved by gene knockdown in combination with a reporter in a wild-type mouse or by electroporation of an expression vector which drives expression of CRE and a reporter into a mouse carrying a conditional floxed allele. In this paradigm one can mainly dissect the cell-autonomous gene function in the targeted neurons, although the presence of non-cell-autonomous effects provided by the wild-type environment will be present (mutant cells in wild-type environment). To investigate non-cell-autonomous effects, it is necessary to electroporate of a separate set of tissue only with the fluorescent reporter in an otherwise mutant environment (mutant cells in mutant environment). Thus, neurons mutant for the same gene in two different environments allows for the distinction of non-cell-autonomous effects, provided that a different phenotype is observed between the mutant cells in each specific environment. Wild-type neurons in an otherwise mutant background by (over)expression of a rescue construct would further allow determination of non-cell-autonomous effects originating from the mutant environment (wild-type cells in mutant environment). The comparison of these three distinct paradigms will facilitate detailed description of cell-autonomous gene function and non-cell-autonomous effects. **(D)** Consecutive electroporation. Consecutive electroporation enables labeling, genetic manipulation and the monitoring of two or more distinct neuronal populations in the developing embryonic brain. The first neuronal population is electroporated for gene knockdown and the consecutive population with control fluorescent markers or vice versa (first mutant, then wild-type). In such assay, the phenotype of the first cohort of electroporated cells can reflect cell-autonomous gene function whereas the phenotype of the second cohort of cells could reflect a combination of directed non-cell-autonomous effects originating from the first cohort and more global community effects. **(E)** MADM. Mosaic analysis with double markers (MADM) allows for the analysis of sparse genetic mosaic (sparse mosaic) versus global/whole tissue (full-KO) ablation of a candidate gene with single cell resolution. This allows to quantitatively analyze non-cell-autonomous effects by subtracting the phenotype present in the sparse mosaic from the full-KO (cell-autonomous + non-cell-autonomous) versus cell-autonomous (sparse mosaic). It is important to note that the background cells in a MADM sparse mosaic are heterozygous and may need adjustment of the paradigm in the case of investigation of a dosage-sensitive gene (haploinsufficiency). In that case, the MADM experiment can also provide a solution by comparing all genotypes/colors, e.g., green –/–, red +/+ and yellow ±. For details of such application the reader is referred to Hippenmeyer et al., 2010. **(F)** Optogenetics. Optogenetics facilitates the use of genetically encoded tools to temporally control gene expression or protein function with light. Viral infection approaches and transgenic mice expressing optogenetic effector proteins in a Cre-dependent manner can be utilized to generate photoactivatable tissue. These approaches can create experimental paradigms which enable investigation of mutant neurons in an otherwise wild-type environment vs. wild-type neurons in a mutant environment in a spatiotemporal manner **(G)** *In toto* imaging. *In toto* live-imaging can visualize the movement of individual cells and their interactions with the surrounding cells within the whole developing tissue. This would allow for a direct assessment of non-cell-autonomous effects exerted by the neighboring cells on an individual cell or *vice versa*. *In toto* imaging mostly involves labeling of all cell membranes so each cell in the organism/microenvironment can be tracked and segmented. Here, a two-color combination of a membrane-localized fluorescent protein and a histone-fused fluorescent protein labeling chromatin which allows for tracking the cell membrane morphologies and nuclei movement has been displayed. Tracking the exact cell boundaries of the neurons spatiotemporally would enable the mapping of the physical interactions and forces which are exerted by the individual cell and that of the surrounding cells.

A chimera is an animal that has two or more populations of genetically distinct cells. Therefore, chimeric animals allow for the presence of mutant cells in an otherwise wild-type background or *vice versa.* Depending on the degree of chimerism (i.e., ratio of wild-type versus mutant cells) such assay offers one way to distinguish between cell-autonomous gene function and non-cell-autonomous mechanisms *in vivo* (Figure 2A) (Gilmore and Herrup, 2001; Hammond et al., 2001; Hammond, 2004). Any phenotypic difference seen between the neurons of the same genotype, but present in distinct genotypic environments indicate non-cell-autonomous effects. However, the degree of chimerism is hard to control, especially in the embryo. Therefore comparative studies across distinct individual animals may be challenging. Yet, a few studies have very successfully applied chimeras to study radial neuronal migration in the cerebral cortex and have described the presence of non-cell-autonomous effects (Hammond et al., 2001; Yang et al., 2002; Hammond, 2004).

### Viral Infection

*In utero* injection of virus encoding a reporter, e.g., green fluorescent protein (GFP) has widely been used to investigate neuronal migration, lineage tracing and clonal analysis *in vivo* (Gaiano et al., 1999; Malatesta et al., 2000; Kaspar et al., 2002; Gupta et al., 2003; Sanada et al., 2004; Stott and Kirik, 2006; He et al., 2015). Retroviral encoding allows to sparsely target developing neurons by either expression of the reporter only (e.g., in a wild-type or mutant environment) or using a virus vector that encodes a wild-type or mutant gene of interest in combination with a reporter. This facilitates the inactivation or rescue of the gene of interest in either wild-type or mutant environments, allowing for the distinction of cell-autonomous gene function and non-cell-autonomous effects (Figure 2B). Appropriate dilution of the retrovirus titer and thus lowering infection rate allows for the discrimination of individual neurons and one can generate more or less sparsely targeted neuronal populations (Noctor et al., 2001; Sanada et al., 2004). In addition, delivery of an adeno-associated virus (AAV) encoding a fluorescent protein and Cre recombinase in combination with a reporter mouse carrying a conditional floxed allele of a candidate gene of interest, can be also be used to target a specific population of neurons (Kaspar et al., 2002). Generally, any approach using a virus which can infect the cell population of interest, achieve specific stable gene expression and reporter labeling can be used to create paradigms for studying cell-autonomous gene function and non-cell-autonomous effects in radial projection neuron migration.

### *In utero* Injection and Electroporation

Timed *in utero* electroporation for inactivation of a gene allows for the sparse targeting of developing neurons in an otherwise wild-type environment (Figure 2C). The inactivation of a specific gene can either be achieved by electroporation of shRNA or miRNA for gene knockdown, in combination with a reporter in a wild-type animal. Alternatively, electroporation of an expression vector which drives expression of CRE and a reporter in a mouse carrying a conditional floxed allele of a candidate gene of interest, can be used (Franco et al., 2011). These paradigms permit the dissection and analysis of cell-autonomous gene function in the targeted neurons. However, the presence of non-cell-autonomous effects originating from the wild-type environment may be present but not easily visualized (Figure 2C). Most studies so far have used this paradigm to study cell-autonomous gene function (Franco et al., 2011; Jossin and Cooper, 2011; Szczurkowska et al., 2018; Kon et al., 2019). To investigate non-cell-autonomous effects and mechanisms one would also need a separate set of tissue only electroporated with the fluorescent reporter to sparsely label the already mutant neurons in an otherwise non-labeled mutant environment (Figure 2C). Having neurons mutant for the same gene in two different environments would allow for the distinction of non-cell-autonomous mechanisms, provided that a different phenotype is observed between the mutant cells in each specific environment. In addition, having wild-type neurons in an otherwise mutant background by (over)expression of a rescue construct would further permit the determination of non-cell-autonomous effects originating from the mutant environment (Figure 2C). Only a few studies have applied this paradigm of rescuing a few cells sparsely in a mutant environment (Gupta et al., 2003; Sanada et al., 2004). Similar to chimeras (Figure 2A), it is important to consider the ratio of the mutant versus wild-type cells. For instance, sparse electroporation allows for the investigation of the direct interaction of cells of distinct genotypes. However, generating a high amount of mutant cells within the wild-type environment might create a local mutant microenvironment where specific interactions between mutant cells could dominate. As a consequence, the presence of a local mutant microenvironment would make it difficult to distinguish cell-autonomous from non-cell-autonomous responses. Therefore, the amount of the electroporated cells should be considered carefully. While sparse single cell deletion of a candidate gene may truly report cell-autonomy of gene function, progressive local increase in the number of mutant cells may lead to a sweet spot from which onward non-cell-autonomous community effects emerge (Nakagawa et al., 2019). Another way of generating very sparse populations of cells using this method, is to transplant micro dissected mutant cells from electroporated corticies to either another wild-type or mutant brain by intraventricular injection (Elias et al., 2007).

Consecutive electroporation enables cellular labeling, genetic manipulation and the monitoring of two or more distinct neuronal populations in the developing embryonic brain (Figure 2D). The first neuronal population could be electroporated for gene knockdown and the consecutive population with control fluorescent markers or vice versa. In such assay, the phenotype of the first cohort of electroporated cells can reflect cell-autonomous gene function whereas the phenotype of the second cohort of cells could reflect a combination of directed non-cell-autonomous cues originating from the first cohort and more global community effects (Jossin and Cooper, 2011; Gil-Sanz et al., 2013; Baek et al., 2015; Greenman et al., 2015).

In summary, sparse *in utero* electroporation for gene knockdown or CRE-dependent conditional gene inactivation in combination with fluorescent reporters facilitates the comparison of mutant phenotypes in distinct cellular environments. Such comparative studies, in principle, enable the systematic dissection of cell-autonomous gene function and/or phenotypes in response to gene inactivation and non-cell-autonomous mechanisms in radial neuronal migration.

### Mosaic Analysis With Double Markers (MADM)

Mosaic analysis with double markers (MADM) technology allows for the analysis of sparse genetic mosaic (sparse mosaic) versus global/whole tissue (full-KO) ablation of a candidate gene, and with single cell resolution (Figure 2E) (Zong et al., 2005; Hippenmeyer et al., 2010; Beattie et al., 2017; Laukoter et al., 2020). Therefore MADM provides a unique genetic tool to investigate cell-autonomous gene functions and the relative contribution of non-cell-autonomous effects. By using MADM one can quantitatively analyze these effects (Figure 2E) (Youn et al., 2009; Hippenmeyer et al., 2010; Hippenmeyer, 2014). In the sparse mosaic animals, mutant neurons are surrounded by ‘normal’ neurons and therefore mainly provide information about cell-autonomous gene function. In addition, the presence of non-cell-autonomous effects originating from the ‘normal’ environment may be present but not easily measured. In the full-knockout of a particular candidate gene, mutant neurons are surrounded by other mutant neurons, and it is not straightforward to distinguish between cell-autonomous gene function and non-cell-autonomous effects. However, one could quantitatively deduct non-cell-autonomous effects by subtracting the phenotype present in the sparse mosaic from the full/cKO (cell-autonomous + non-cell-autonomous versus cell-autonomous (sparse mosaic) (Figure 2E). The sparse mosaic versus full/cKO paradigm thus offers a promising experimental platform to investigate non-cell-autonomous effects because any phenotypic differences observed when the two paradigms are compared can be quantitatively assessed at single cell resolution (Beattie et al., 2017; Laukoter et al., 2020). Nevertheless, generating a full-knockout where all cells are mutant for a particular candidate gene can be problematic since many migration genes are lethal when knocked out completely (Hirotsune et al., 1998; Sasaki et al., 2005). Conditional-knockout mice could be analyzed, provided that floxed alleles are available. In the future, systematic assay of almost any candidate gene will be in principle enabled by the whole-genome MADM library resource (Contreras et al., 2020).

### Optogenetics

Optogenetics facilitates the use of genetically encoded tools to temporally control gene expression or protein function with light. It can facilitate localized modifications spatiotemporally within living cells and animals, targeting a wide array of proteins, e.g., involved in cell-migration, cell–cell adhesion, and force transduction (Guglielmi et al., 2016; Mühlhäuser et al., 2017). Using this method one can investigate how changes in individual cells influence neighboring cells and global tissue remodeling. So far, most experiments applying optogenetics for studying cell-migration have mainly been applied to *in vitro* cell culture systems and small *in vivo* systems, e.g., during gastrulation (Wang et al., 2010; Kim et al., 2014; Weitzman and Hahn, 2014; Valon et al., 2017; Wang and Cooper, 2017). In the mouse brain, optogenetics have mainly been used to activate, inhibit, or detect neuronal activity (Montagni et al., 2019). However, spatiotemporal control of the expression of a candidate gene or the activity of a specific signaling pathway could provide valuable insights into the dissection of non-cell-autonomous mechanisms in projection neuron migration. Currently, various viral infection approaches and transgenic mice expressing optogenetic effector proteins in a Cre-dependent manner can be utilized to generate photoactivatable cells and tissue (Madisen et al., 2012; Guglielmi et al., 2016). These approaches can create experimental paradigms similar to the ones described above (mutant neuron in an otherwise wild-type environment vs. wild-type neurons in a mutant environment) (Figure 2F), however, with spatiotemporal control of gene expression or protein function. This would allow exact targeting of specific neurons at specific sequential steps along the migratory path. In addition, one would be able to perturb cells of a specific cohort to see the exact non-autonomous effects on the surrounding non-stimulated neurons (Figure 2F). An interesting aspect for which an optogenetic approach could also provide information is to what extent the ratio of mutant and wild-type cells in the same tissue is needed to see non-cell-autonomous effects. Starting from targeting only one cell and then increasing the area which is activated by light stimulation could reveal the threshold for when non-cell-autonomous mechanisms emerge dependent on the cell ratio of mutant vs. wild-type present. However, for *in vivo* and *in situ* experiments of mouse tissue, such an optogenetic approach might prove technically difficult. Activating one specific moving cell or a certain area of the tissue with a beam of light can be quite difficult *in vivo* and in three-dimensional intact tissues.

### *In toto* Live-Imaging

*In toto* live-imaging can visualize the movement of individual cells and their interactions with the surrounding cells within the whole developing tissue (Megason and Fraser, 2007; Veeman and Reeves, 2015; McDole et al., 2018). This would enable a direct assessment of non-cell-autonomous effects exerted by the neighboring cells on an individual cell or *vice versa*. So far, this method has mostly been used to visualize cell and collective migration behaviors in smaller *in vivo* systems such as, e.g., *Drosophila* (Krzic et al., 2012; Tomer et al., 2012), zebrafish (Nogare et al., 2017; Hiscock et al., 2018; Shah et al., 2019) and larger systems such as mouse gastrulation and heart tissue (Megason and Fraser, 2007; Stewart et al., 2009; McDole et al., 2018; Yue et al., 2020). *In toto* imaging mostly involves labeling of all cell membranes so each cell in the organism/microenvironment can be tracked and segmented (Nogare et al., 2017). In addition, both the cell membranes and the cell nuclei can be labeled in two individual colors for a more precise segmentation which does not rely on estimation. For *in vivo* studies of embryogenesis, a two-color combination of a membrane-localized fluorescent protein and a histone-fused fluorescent protein labeling chromatin enables the tracking of the cell membrane morphologies and nuclei movements (Megason, 2009; Stewart et al., 2009). Tracking all cells in an area of interest and their physical interactions would allow for a much more detailed analysis of the cellular dynamics which are ongoing during neuronal migration (Figure 2G). Achieving a resolution in which the exact cell boundaries of the neurons could be tracked spatiotemporally would enable the mapping of the physical interactions and forces which are exerted by the individual cell and that of the surrounding cells. Such mapping could help understand where and when certain cell dynamics are being subjected to non-cell-autonomous forces that evoke a response in the individual cell from the surrounding environment or *vice versa*. Future development of imaging approaches like, e.g., light-sheet microscopy could facilitate the spatiotemporal resolution needed to visualize the migration of interacting neighboring cells in bigger tissues like the mouse cerebral cortex.

## Outlook

Non-cell-autonomous mechanisms play an important role during brain development. However, little is known about the exact nature and physiological function of these non-autonomous mechanisms in radial neuronal migration. Thus, a number of open key aspects and questions require attention in future investigations. First, how can non-cell-autonomous mechanisms be distinguished from cell-autonomous cues and intrinsic gene function? Second, how can non-cell-autonomous effects be quantified and the underlying mechanisms determined? Third, what role do non-cell-autonomous mechanisms play in disease? Focal malformations of cortical development (FMCD) represent one example of a disorder where a localized cortical lesion, i.e., mutations in a small fraction of cells, disrupts the entire cortical architecture. In the most severe cases, devastating pediatric hemimegalencephaly may emerge, which is characterized by enlargement of one entire cerebral cortex hemisphere (Lee et al., 2012; Poduri et al., 2012, 2013; Rivière et al., 2012). Hence, it is also important from a clinical perspective to precisely dissect the contribution of non-cell-autonomous, tissue-wide and systemic mechanisms in cortical development in general and neuronal migration in particular. The better understanding of the interplay of cell intrinsic gene function and non-cell-autonomous effects will enable further comprehension of the underlying etiology of neurodevelopmental disorders due to genetic mutations (Guerrini et al., 2008; Guerrini and Parrini, 2010).

## Author Contributions

Both authors listed have made a substantial, direct and intellectual contribution to the work, and approved it for publication.

## Conflict of Interest

The authors declare that the research was conducted in the absence of any commercial or financial relationships that could be construed as a potential conflict of interest.

## References

[B1] Allendoerfer K. L. Shatz C. J. (1994). The subplate, a transient neocortical structure: its role in the development of connections between thalamus and cortex. *Annu. Rev. Neurosci.* 17 185–218. 10.1146/annurev.ne.17.030194.001153 8210173

[B2] Angevine J. B. Sidman R. L. (1961). Autoradiographic study of cell migration during histogenesis of cerebral cortex in the mouse. *Nature* 192 766–768. 10.1038/192766b0 17533671

[B3] Ayala R. Shu T. Tsai L. H. (2007). Trekking across the brain: the journey of neuronal migration. *Cell* 128 29–43. 10.1016/j.cell.2006.12.021 17218253

[B4] Baek S. T. Copeland B. Yun E.-J. Kwon S.-K. Guemez-Gamboa A. Schaffer A. E. (2015). An AKT3-FOXG1-reelin network underlies defective migration in human focal malformations of cortical development. *Nat. Med.* 21 1445–1454. 10.1038/nm.3982 26523971 PMC4955611

[B5] Barros C. S. Franco S. J. Muller U. (2011). Extracellular matrix: functions in the nervous system. *Cold Spring Harb. Perspect. Biol.* 3:a005108. 10.1101/cshperspect.a005108 21123393 PMC3003458

[B6] Beattie R. Postiglione M. P. Burnett L. E. Laukoter S. Streicher C. Pauler F. M. (2017). Mosaic analysis with double markers reveals distinct sequential functions of Lgl1 in neural stem cells. *Neuron* 94 517–533.e3. 28472654 10.1016/j.neuron.2017.04.012

[B7] Becker S. F. S. Mayor R. Kashef J. (2013). Cadherin-11 mediates contact inhibition of locomotion during *Xenopus* neural crest cell migration. *PLoS One* 8:e85717. 10.1371/journal.pone.0085717 24392028 PMC3877381

[B8] Belvindrah R. Graus-Porta D. Goebbels S. Nave K.-A. Muller U. (2007). 1 integrins in radial glia but not in migrating neurons are essential for the formation of cell layers in the cerebral cortex. *J. Neurosci.* 27 13854–13865. 10.1523/jneurosci.4494-07.2007 18077697 PMC6673609

[B9] Borrell V. Götz M. (2014). Role of radial glial cells in cerebral cortex folding. *Curr. Opin. Neurobiol.* 27 39–46. 10.1016/j.conb.2014.02.007 24632307

[B10] Cappello S. Böhringer C. R. J. Bergami M. Conzelmann K. K. Ghanem A. Tomassy G. S. (2012). A radial glia-specific role of RhoA in double cortex formation. *Neuron* 73 911–924. 10.1016/j.neuron.2011.12.030 22405202

[B11] Caviness V. S. Sidman R. L. (1973). Time of origin or corresponding cell classes in the cerebral cortex of normal and reeler mutant mice: an autoradiographic analysis. *J. Comp. Neurol.* 148 141–151. 10.1002/cne.901480202 4700506

[B12] Contreras X. Davaatseren A. Amberg N. Hansen A. H. Sonntag J. Andersen L. (2020). A genome-wide library of MADM mice for single-cell genetic mosaic analysis. *BioRxiv [Preprint]* 10.1101/2020.06.05.136192 PMC8317686 34161767

[B13] D’Arcangelo G. (2014). Reelin in the years: controlling neuronal migration and maturation in the mammalian brain. *Adv. Neurosci.* 2014:597395.

[B14] del Toro D. Carrasquero-Ordaz M. A. Chu A. Ruff T. Shahin M. Jackson V. A. (2020). Structural basis of teneurin-latrophilin interaction in repulsive guidance of migrating neurons. *Cell* 180 323–339.e19. 31928845 10.1016/j.cell.2019.12.014 PMC6978801

[B15] del Toro D. Ruff T. Cederfjäll E. Villalba A. Seyit-Bremer G. Borrell V. (2017). Regulation of cerebral cortex folding by controlling neuronal migration via FLRT adhesion molecules. *Cell* 169 621–635.e16. 28475893 10.1016/j.cell.2017.04.012

[B16] Elias L. A. Wang D. D. Kriegstein A. R. (2007). Gap junction adhesion is necessary for radial migration in the neocortex. *Nature* 448 901–907. 10.1038/nature06063 17713529

[B17] Evsyukova I. Plestant C. Anton E. S. (2013). Integrative mechanisms of oriented neuronal migration in the developing brain. *Annu. Rev. Cell Dev. Biol.* 29 299–353. 10.1146/annurev-cellbio-101512-122400 23937349 PMC3930923

[B18] Ford-Perriss M. Abud H. Murphy M. (2001). Fibroblast growth factors in the developing central nervous system. *Clin. Exp. Pharmacol. Physiol.* 28 493–503. 10.1046/j.1440-1681.2001.03477.x 11422214

[B19] Franco S. J. Martinez-Garay I. Gil-Sanz C. Harkins-Perry S. R. Müller U. (2011). Reelin regulates cadherin function via Dab1/Rap1 to control neuronal migration and lamination in the neocortex. *Neuron* 69 482–497. 10.1016/j.neuron.2011.01.003 21315259 PMC3056352

[B20] Franco S. J. Müller U. (2011). Extracellular matrix functions during neuronal migration and lamination in the mammalian central nervous system. *Dev. Neurobiol.* 71 889–900. 10.1002/dneu.20946 21739613 PMC3490208

[B21] Friedl P. Gilmour D. (2009). Collective cell migration in morphogenesis, regeneration and cancer. *Nat. Rev. Mol. Cell Biol.* 10 445–457. 10.1038/nrm2720 19546857

[B22] Gaiano N. Kohtz J. D. Turnbull D. H. Fishell G. (1999). A method for rapid gain-of-function studies in the mouse embryonic nervous system. *Nat. Neurosci.* 2 812–819. 10.1038/12186 10461220

[B23] Gilmore E. C. Herrup K. (2001). Neocortical cell migration: GABAergic neurons and cells in layers I and VI move in a cyclin-dependent kinase 5-independent manner. *J. Neurosci.* 21 9690–9700. 10.1523/jneurosci.21-24-09690.2001 11739578 PMC6763059

[B24] Gil-Sanz C. Franco S. J. Martinez-Garay I. Espinosa A. Harkins-Perry S. Müller U. (2013). Cajal-retzius cells instruct neuronal migration by coincidence signaling between secreted and contact-dependent guidance cues. *Neuron* 79 461–477. 10.1016/j.neuron.2013.06.040 23931996 PMC3774067

[B25] Gleeson J. G. Walsh C. A. (2000). Neuronal migration disorders: from genetic diseases to developmental mechanisms. *Trends Neurosci.* 23 352–359. 10.1016/s0166-2236(00)01607-6 10906798

[B26] Gorelik A. Sapir T. Woodruff T. M. Reiner O. (2017). *Serping1*/C1 inhibitor affects cortical development in a cell autonomous and non-cell autonomous manner. *Front. Cell. Neurosci.* 11:169. 10.3389/fncel.2017.00169 28670268 PMC5472692

[B27] Graus-Porta D. Blaess S. Senften M. Littlewood-Evans A. Damsky C. Huang Z. (2001). β1-Class integrins regulate the development of laminae and folia in the cerebral and cerebellar cortex. *Neuron* 31 367–379. 10.1016/s0896-6273(01)00374-9 11516395

[B28] Greenman R. Gorelik A. Sapir T. Baumgart J. Zamor V. Segal-Salto M. (2015). Non-cell autonomous and non-catalytic activities of ATX in the developing brain. *Front. Neurosci.* 9:53. 10.3389/fnins.2015.00053 25788872 PMC4349085

[B29] Greig L. C. Woodworth M. B. Galazo M. J. Padmanabhan H. Macklis J. D. (2013). Molecular logic of neocortical projection neuron specification, development and diversity. *Nat. Rev. Neurosci.* 14 755–769. 10.1038/nrn3586 24105342 PMC3876965

[B30] Guerrini R. Dobyns W. B. Barkovich A. J. (2008). Abnormal development of the human cerebral cortex: genetics, functional consequences and treatment options. *Trends Neurosci.* 31 154–162. 10.1016/j.tins.2007.12.004 18262290

[B31] Guerrini R. Parrini E. (2010). Neuronal migration disorders. *Neurobiol. Dis.* 38 154–166. 19245832 10.1016/j.nbd.2009.02.008

[B32] Guglielmi G. Falk H. J. De Renzis S. (2016). Optogenetic control of protein function: from intracellular processes to tissue morphogenesis. *Trends Cell Biol.* 26 864–874. 10.1016/j.tcb.2016.09.006 27727011 PMC5080449

[B33] Gupta A. Sanada K. Miyamoto D. T. Rovelstad S. Nadarajah B. Pearlman A. L. (2003). Layering defect in p35 deficiency is linked to improper neuronal-glial interaction in radial migration. *Nat. Neurosci.* 6 1284–1291. 10.1038/nn1151 14608361

[B34] Hammond V. (2004). Control of cortical neuron migration and layering: cell and non cell-autonomous effects of p35. *J. Neurosci.* 24 576–587. 10.1523/jneurosci.4529-03.2004 14724258 PMC6729984

[B35] Hammond V. Howell B. Godinho L. Tan S. S. (2001). Disabled-1 functions cell autonomously during radial migration and cortical layering of pyramidal neurons. *J. Neurosci.* 21 8798–8808. 10.1523/jneurosci.21-22-08798.2001 11698592 PMC6762297

[B36] Hansen A. H. Duellberg C. Mieck C. Loose M. Hippenmeyer S. (2017). Cell polarity in cerebral cortex development—cellular architecture shaped by biochemical networks. *Front. Cell. Neurosci.* 11:176. 10.3389/fncel.2017.00176 28701923 PMC5487411

[B37] Hansen D. V. Lui J. H. Parker P. R. L. Kriegstein A. R. (2010). Neurogenic radial glia in the outer subventricular zone of human neocortex. *Nature* 464 554–561. 10.1038/nature08845 20154730

[B38] Hatanaka Y. Hisanaga S. I. Heizmann C. W. Murakami F. (2004). Distinct migratory behavior of early- and late-born neurons derived from the cortical ventricular zone. *J. Comp. Neurol.* 479 1–14. 10.1002/cne.20256 15389616

[B39] Hatanaka Y. Zhu Y. Torigoe M. Kita Y. Murakami F. (2016). From migration to settlement: the pathways, migration modes and dynamics of neurons in the developing brain. *Proc. Jpn. Acad. Ser. B Phys. Biol. Sci.* 92 1–19. 10.2183/pjab.92.1 26755396 PMC4880546

[B40] He S. Li Z. Ge S. Yu Y.-C. Shi S.-H. (2015). Inside-out radial migration facilitates lineage-dependent neocortical microcircuit assembly. *Neuron* 86 1159–1166. 10.1016/j.neuron.2015.05.002 26050035 PMC4458701

[B41] Heng J. I. T. Chariot A. Nguyen L. (2010). Molecular layers underlying cytoskeletal remodelling during cortical development. *Trends Neurosci.* 33 38–47. 10.1016/j.tins.2009.09.003 19837469

[B42] Hippenmeyer S. (2014). Cellular and molecular control of neuronal migration. *Adv. Exp. Med. Biol.* 800 1–24. 10.1007/978-94-007-7687-6_1 24243097

[B43] Hippenmeyer S. Youn Y. H. Moon H. M. Miyamichi K. Zong H. Wynshaw-Boris A. (2010). Genetic mosaic dissection of Lis1 and Ndel1 in neuronal migration. *Neuron* 68 695–709. 10.1016/j.neuron.2010.09.027 21092859 PMC3044607

[B44] Hirotsune S. Fleck M. W. Gambello M. J. Bix G. J. Chen A. Clark G. D. (1998). Graded reduction of Pafah1b1 (Lis1) activity results in neuronal migration defects and early embryonic lethality. *Nat. Genet.* 19 333–339. 10.1038/1221 9697693

[B45] Hiscock T. W. Miesfeld J. B. Mosaliganti K. R. Link B. A. Megason S. G. (2018). Feedback between tissue packing and neurogenesis in the zebrafish neural tube. *Development* 145:dev157040. 10.1242/dev.157040 29678815 PMC5992593

[B46] Honda T. Kobayashi K. Mikoshiba K. Nakajima K. (2011). Regulation of cortical neuron migration by the reelin signaling pathway. *Neurochem. Res.* 36 1270–1279. 10.1007/s11064-011-0407-4 21253854

[B47] Huttenlocher A. Lakonishok M. Kinder M. Wu S. Truong T. Knudsen K. A. (1998). Integrin and cadherin synergy regulates contact inhibition of migration and motile activity. *J. Cell Biol.* 141 515–526. 10.1083/jcb.141.2.515 9548728 PMC2148455

[B48] Jossin Y. (2020). Molecular mechanisms of cell polarity in a range of model systems and in migrating neurons. *Mol. Cell. Neurosci.* 106:103503. 10.1016/j.mcn.2020.103503 32485296

[B49] Jossin Y. Cooper J. A. (2011). Reelin, Rap1 and N-cadherin orient the migration of multipolar neurons in the developing neocortex. *Nat. Neurosci.* 14 697–703. 10.1038/nn.2816 21516100 PMC3102785

[B50] Jossin Y. Gui L. Goffinet A. M. (2007). Processing of Reelin by embryonic neurons is important for function in tissue but not in dissociated cultured neurons. *J. Neurosci.* 27 4243–4252. 10.1523/jneurosci.0023-07.2007 17442808 PMC6672330

[B51] Kaspar B. K. Vissel B. Bengoechea T. Crone S. Randolph-Moore L. Muller R. (2002). Adeno-associated virus effectively mediates conditional gene modification in the brain. *Proc. Natl. Acad. Sci. U.S.A.* 99 2320–2325. 10.1073/pnas.042678699 11842206 PMC122363

[B52] Kawauchi T. (2012). Cell adhesion and its endocytic regulation in cell migration during neural development and cancer metastasis. *Int. J. Mol. Sci.* 13 4564–4590. 10.3390/ijms13044564 22605996 PMC3344232

[B53] Kawauchi T. (2014). Cdk5 regulates multiple cellular events in neural development, function and disease. *Dev. Growth Differ.* 56 335–348. 10.1111/dgd.12138 24844647

[B54] Kawauchi T. (2015). Cellullar insights into cerebral cortical development: focusing on the locomotion mode of neuronal migration. *Front. Cell. Neurosci.* 9:394. 10.3389/fncel.2015.00394 26500496 PMC4595654

[B55] Kawauchi T. Sekine K. Shikanai M. Chihama K. Tomita K. Kubo K. I. (2010). Rab GTPases-dependent endocytic pathways regulate neuronal migration and maturation through N-cadherin trafficking. *Neuron* 67 588–602. 10.1016/j.neuron.2010.07.007 20797536

[B56] Kim N. Kim J. M. Lee M. Kim C. Y. Chang K. Y. Heo W. D. (2014). Spatiotemporal control of fibroblast growth factor receptor signals by blue light. *Chem. Biol.* 21 903–912. 10.1016/j.chembiol.2014.05.013 24981772

[B57] Koie M. Okumura K. Hisanaga A. Kamei T. Sasaki K. Deng M. (2014). Cleavage within reelin repeat 3 regulates the duration and range of the signaling activity of reelin protein. *J. Biol. Chem.* 289 12922–12930. 10.1074/jbc.m113.536326 24644294 PMC4007479

[B58] Kon E. Calvo-Jiménez E. Cossard A. Na Y. Cooper J. A. Jossin Y. (2019). N-cadherin-regulated FGFR ubiquitination and degradation control mammalian neocortical projection neuron migration. *eLife* 8:e47673. 10.7554/eLife.47673 PMC6786859 31577229

[B59] Kriegstein A. R. Noctor S. C. (2004). Patterns of neuronal migration in the embryonic cortex. *Trends Neurosci.* 27 392–399. 10.1016/j.tins.2004.05.001 15219738

[B60] Krzic U. Gunther S. Saunders T. E. Streichan S. J. Hufnagel L. (2012). Multiview light-sheet microscope for rapid in toto imaging. *Nat. Methods* 9 730–733. 10.1038/nmeth.2064 22660739

[B61] Kubo K.-I. Honda T. Tomita K. Sekine K. Ishii K. Uto A. (2010). Ectopic reelin induces neuronal aggregation with a normal birthdate-dependent “inside-out” alignment in the developing neocortex. *J. Neurosci.* 30 10953–10966. 10.1523/jneurosci.0486-10.2010 20720102 PMC6633482

[B62] Kwan K. Y. Sestan N. Anton E. S. (2012). Transcriptional co-regulation of neuronal migration and laminar identity in the neocortex. *Development* 139 1535–1546. 10.1242/dev.069963 22492350 PMC3317962

[B63] Kwon Y. T. Gupta A. Zhou Y. Nikolic M. Tsai L. H. (2000). Regulation of N-cadherin-mediated adhesion by the p35-Cdk5 kinase. *Curr. Biol.* 10 363–372. 10.1016/s0960-9822(00)00411-5 10753743

[B64] Laukoter S. Beattie R. Pauler F. M. Amberg N. Nakayama K. I. Hippenmeyer S. (2020). Imprinted Cdkn1c genomic locus cell-autonomously promotes cell survival in cerebral cortex development. *Nat. Commun.* 11:195. 10.1038/s41467-019-14077-2 PMC6954230 31924768

[B65] Lee J. H. Huynh M. Silhavy J. L. Kim S. Dixon-Salazar T. Heiberg A. (2012). De novo somatic mutations in components of the PI3K-AKT3-mTOR pathway cause hemimegalencephaly. *Nat. Genet.* 44 941–945. 10.1038/ng.2329 22729223 PMC4417942

[B66] Lodato S. Arlotta P. (2015). Generating neuronal diversity in the mammalian cerebral cortex. *Annu. Rev. Cell Dev. Biol.* 31 699–720. 10.1146/annurev-cellbio-100814-125353 26359774 PMC4778709

[B67] Londono C. Loureiro M. J. Slater B. Lücker P. B. Soleas J. Sathananthan S. (2014). Nonautonomous contact guidance signaling during collective cell migration. *Proc. Natl. Acad. Sci. U.S.A.* 111 1807–1812. 10.1073/pnas.1321852111 24449852 PMC3918762

[B68] Long K. R. Huttner W. B. (2019). How the extracellular matrix shapes neural development. *Open Biol.* 9:180216. 10.1098/rsob.180216 30958121 PMC6367132

[B69] Madisen L. Mao T. Koch H. Zhuo J. Berenyi A. Fujisawa S. (2012). A toolbox of Cre-dependent optogenetic transgenic mice for light-induced activation and silencing. *Nat. Neurosci.* 15 793–802. 10.1038/nn.3078 22446880 PMC3337962

[B70] Maeda N. (2015). Proteoglycans and neuronal migration in the cerebral cortex during development and disease. *Front. Neurosci.* 9:98. 10.3389/fnins.2015.00098 25852466 PMC4369650

[B71] Malatesta P. Hartfuss E. Götz M. (2000). Isolation of radial glial cells by fluorescent-activated cell sorting reveals a neural lineage. *Development* 127 5253–5263. 11076748 10.1242/dev.127.24.5253

[B72] McConnell S. K. (1995). Constructing the cerebral cortex: neurogenesis and fate determination. *Neuron* 15 761–768. 10.1016/0896-6273(95)90168-x 7576626

[B73] McDole K. Guignard L. Amat F. Berger A. Malandain G. Royer L. A. (2018). In toto imaging and reconstruction of post-implantation mouse development at the single-cell level. *Cell* 175 859–876.e33. 30318151 10.1016/j.cell.2018.09.031

[B74] Megason S. G. (2009). “In toto imaging of embryogenesis with confocal time-lapse microscopy,” in *Zebrafish: Methods and Protocols*, eds Lieschke G. J. Oates A. C. Kawakami K. (Totowa, NJ: Humana Press), 317–332. 10.1007/978-1-60327-977-2_19 PMC2826616 19378112

[B75] Megason S. G. Fraser S. E. (2007). Imaging in systems biology. *Cell* 130 784–795. 17803903 10.1016/j.cell.2007.08.031

[B76] Montagni E. Resta F. Mascaro A. L. A. Pavone F. S. (2019). Optogenetics in brain research: from a strategy to investigate physiological function to a therapeutic tool. *Photonics* 6:92 10.3390/photonics6030092

[B77] Mühlhäuser W. W. D. Fischer A. Weber W. Radziwill G. (2017). Optogenetics - bringing light into the darkness of mammalian signal transduction. *Biochim. Biophys. Acta Mol. Cell Res.* 1864 280–292. 27845208 10.1016/j.bbamcr.2016.11.009

[B78] Nadarajah B. Alifragis P. Wong R. O. L. Parnavelas J. G. (2003). Neuronal migration in the developing cerebral cortex: observations based on real-time imaging. *Cereb. Cortex* 13 607–611. 10.1093/cercor/13.6.607 12764035

[B79] Nadarajah B. Brunstrom J. E. Grutzendler J. Wong R. O. Pearlman A. L. (2001). Two modes of radial migration in early development of the cerebral cortex. *Nat. Neurosci.* 4 143–150. 10.1038/83967 11175874

[B80] Nakagawa N. Plestant C. Yabuno-Nakagawa K. Li J. Lee J. Huang C.-W. (2019). Memo1-mediated tiling of radial glial cells facilitates cerebral cortical development. *Neuron* 103 836–852.e5. 31277925 10.1016/j.neuron.2019.05.049 PMC6728225

[B81] Noctor S. C. Flint A. C. Weissman T. A. Dammerman R. S. Kriegstein A. R. (2001). Neurons derived from radial glial cells establish radial units in neocortex. *Nature* 409 714–720. 10.1038/35055553 11217860

[B82] Noctor S. C. Martínez-Cerdeño V. Ivic L. Kriegstein A. R. (2004). Cortical neurons arise in symmetric and asymmetric division zones and migrate through specific phases. *Nat. Neurosci.* 7 136–144. 10.1038/nn1172 14703572

[B83] Nogare D. D. Nikaido M. Somers K. Head J. Piotrowski T. Chitnis A. B. (2017). In toto imaging of the migrating Zebrafish lateral line primordium at single cell resolution. *Dev. Biol.* 422 14–23. 10.1016/j.ydbio.2016.12.015 27965055

[B84] Ogawa M. Miyata T. Nakajima K. Yagyu K. Seike M. Ikenaka K. (1995). The reeler gene-associated antigen on Cajal-Retzius neurons is a crucial molecule for laminar organization of cortical neurons. *Neuron* 14 899–912. 10.1016/0896-6273(95)90329-1 7748558

[B85] Ornitz D. M. Itoh N. (2015). The fibroblast growth factor signaling pathway. *Wiley Interdiscip. Rev. Dev. Biol.* 4 215–266. 10.1002/wdev.176 25772309 PMC4393358

[B86] Poduri A. Evrony G. D. Cai X. Elhosary P. C. Beroukhim R. Lehtinen M. K. (2012). Somatic activation of AKT3 causes hemispheric developmental brain malformations. *Neuron* 74 41–48. 10.1016/j.neuron.2012.03.010 22500628 PMC3460551

[B87] Poduri A. Evrony G. D. Cai X. Walsh C. A. (2013). Somatic mutation, genomic variation, and neurological disease. *Science* 341:1237758. 10.1126/science.1237758 23828942 PMC3909954

[B88] Pramparo T. Libiger O. Jain S. Li H. Youn Y. H. Hirotsune S. (2011). Global developmental gene expression and pathway analysis of normal brain development and mouse models of human neuronal migration defects. *PLoS Genet.* 7:e1001331. 10.1371/journal.pgen.1001331 21423666 PMC3053345

[B89] Rakic P. (1972). Mode of cell migration to the superficial layers of fetal monkey neocortex. *J. Comp. Neurol.* 145 61–83. 10.1002/cne.901450105 4624784

[B90] Rice D. S. Sheldon M. D’Arcangelo G. Nakajima K. Goldowitz D. Curran T. (1998). Disabled-1 acts downstream of Reelin in a signaling pathway that controls laminar organization in the mammalian brain. *Development* 125 3719–3729. 9716537 10.1242/dev.125.18.3719

[B91] Rivière J. B. Mirzaa G. M. O’Roak B. J. Beddaoui M. Alcantara D. Conway R. L. (2012). De novo germline and postzygotic mutations in AKT3, PIK3R2 and PIK3CA cause a spectrum of related megalencephaly syndromes. *Nat. Genet.* 44 934–940. 10.1038/ng.2331 22729224 PMC3408813

[B92] Sanada K. Gupta A. Tsai L. H. (2004). Disabled-1-regulated adhesion of migrating neurons to radial glial fiber contributes to neuronal positioning during early corticogenesis. *Neuron* 42 197–211. 10.1016/s0896-6273(04)00222-3 15091337

[B93] Sasaki S. Mori D. Toyo-oka K. Chen A. Garrett-beal L. Muramatsu M. (2005). Complete loss of Ndel1 results in neuronal migration defects and early embryonic lethality. *Mol. Cell. Biol.* 25 7812–7827. 10.1128/mcb.25.17.7812-7827.2005 16107726 PMC1190282

[B94] Schaar B. T. McConnell S. K. (2005). Cytoskeletal coordination during neuronal migration. *Proc. Natl. Acad. Sci. U.S.A.* 102 13652–13657. 10.1073/pnas.0506008102 16174753 PMC1199551

[B95] Seiradake E. delToro D. Nagel D. Cop F. Härtl R. Ruff T. (2014). FLRT structure: balancing repulsion and cell adhesion in cortical and vascular development. *Neuron* 84 370–385. 10.1016/j.neuron.2014.10.008 25374360 PMC4210639

[B96] Sekine K. Kawauchi T. Kubo K. Honda T. Herz J. Hattori M. (2012). Reelin controls neuronal positioning by promoting cell-matrix adhesion via inside-out activation of integrin α5β1. *Neuron* 76 353–369. 10.1016/j.neuron.2012.07.020 23083738 PMC3479437

[B97] Sekine K. Kubo K. I. Nakajima K. (2014). How does Reelin control neuronal migration and layer formation in the developing mammalian neocortex? *Neurosci. Res.* 86 50–58. 10.1016/j.neures.2014.06.004 24969097

[B98] Shah G. Thierbach K. Schmid B. Waschke J. Reade A. Hlawitschka M. (2019). Multi-scale imaging and analysis identify pan-embryo cell dynamics of germlayer formation in zebrafish. *Nat. Commun.* 10:5753. 10.1038/s41467-019-13625-0 PMC6917746 31848345

[B99] Shellard A. Mayor R. (2020). Rules of collective migration: from the wildebeest to the neural crest. *Philos. Trans. R. Soc. B Biol. Sci.* 375:20190387. 10.1098/rstb.2019.0387 32713298 PMC7423382

[B100] Simo S. Jossin Y. Cooper J. A. (2010). Cullin 5 regulates cortical layering by modulating the speed and duration of Dab1-dependent neuronal migration. *J. Neurosci.* 30 5668–5676. 10.1523/jneurosci.0035-10.2010 20410119 PMC2866641

[B101] Stewart M. D. Jang C.-W. Hong N. W. Austin A. P. Behringer R. R. (2009). Dual fluorescent protein reporters for studying cell behaviors in vivo. *Genesis* 47 708–717. 10.1002/dvg.20565 19813259 PMC3384709

[B102] Stott S. R. W. Kirik D. (2006). Targeted in utero delivery of a retroviral vector for gene transfer in the rodent brain. *Eur. J. Neurosci.* 24 1897–1906. 10.1111/j.1460-9568.2006.05095.x 17067293

[B103] Su S. C. Tsai L. H. (2011). Cyclin-dependent kinases in brain development and disease. *Annu. Rev. Cell Dev. Biol.* 27 465–491. 10.1146/annurev-cellbio-092910-154023 21740229

[B104] Szczurkowska J. Pischedda F. Pinto B. Managò F. Haas C. A. Summa M. (2018). NEGR1 and FGFR2 cooperatively regulate cortical development and core behaviours related to autism disorders in mice. *Brain* 141 2772–2794. 30059965 10.1093/brain/awy190 PMC6113639

[B105] Tabata H. Kanatani S. Nakajima K. (2009). Differences of migratory behavior between direct progeny of apical progenitors and basal progenitors in the developing cerebral cortex. *Cereb. Cortex* 19 2092–2105. 10.1093/cercor/bhn227 19150920

[B106] Tabata H. Nakajima K. (2003). Multipolar migration: the third mode of radial neuronal migration in the developing cerebral cortex. *J. Neurosci.* 23 9996–10001. 10.1523/jneurosci.23-31-09996.2003 14602813 PMC6740853

[B107] Tada M. Heisenberg C.-P. (2012). Convergent extension: using collective cell migration and cell intercalation to shape embryos. *Development* 139 3897–3904. 10.1242/dev.073007 23048180

[B108] Tomer R. Khairy K. Amat F. Keller P. J. (2012). Quantitative high-speed imaging of entire developing embryos with simultaneous multiview light-sheet microscopy. *Nat. Methods* 9 755–763. 10.1038/nmeth.2062 22660741

[B109] Valiente M. Marín O. (2010). Neuronal migration mechanisms in development and disease. *Curr. Opin. Neurobiol.* 20 68–78. 10.1016/j.conb.2009.12.003 20053546

[B110] Valon L. Marín-Llauradó A. Wyatt T. Charras G. Trepat X. (2017). Optogenetic control of cellular forces and mechanotransduction. *Nat. Commun.* 8:14396. 10.1038/ncomms14396 PMC5309899 28186127

[B111] van den Berghe V. Stappers E. Seuntjens E. (2014). How cell-autonomous is neuronal migration in the forebrain? Molecular cross-talk at the cell membrane. *Neuroscientist* 20 571–575. 10.1177/1073858414539396 24972605

[B112] Veeman M. Reeves W. (2015). Quantitative and in toto imaging in ascidians: working toward an image-centric systems biology of chordate morphogenesis. *Genesis* 53 143–159. 10.1002/dvg.22828 25262824 PMC4378666

[B113] Wang L. Cooper J. A. (2017). Optogenetic control of the Dab1 signaling pathway. *Sci. Rep.* 7:43760. 10.1038/srep43760 PMC5363252 28272509

[B114] Wang X. He L. Wu Y. I. Hahn K. M. Montell D. J. (2010). Light-mediated activation reveals a key role for Rac in collective guidance of cell movement in vivo. *Nat. Cell Biol.* 12 591–597. 10.1038/ncb2061 20473296 PMC2929827

[B115] Wang X. Tsai J. W. Lamonica B. Kriegstein A. R. (2011). A new subtype of progenitor cell in the mouse embryonic neocortex. *Nat. Neurosci.* 14 555–562. 10.1038/nn.2807 21478886 PMC3083489

[B116] Weitzman M. Hahn K. M. (2014). Optogenetic approaches to cell migration and beyond. *Curr. Opin. Cell Biol.* 30 112–120. 10.1016/j.ceb.2014.08.004 25216352 PMC4215803

[B117] Yanagida M. Miyoshi R. Toyokuni R. Zhu Y. Murakami F. (2012). Dynamics of the leading process, nucleus, and Golgi apparatus of migrating cortical interneurons in living mouse embryos. *Proc. Natl. Acad. Sci. U.S.A.* 109 16737–16742. 10.1073/pnas.1209166109 23010922 PMC3478636

[B118] Yang H. Jensen P. Goldowitz D. (2002). The community effect and Purkinje cell migration in the cerebellar cortex: analysis of scrambler chimeric mice. *J. Neurosci.* 22 464–470. 10.1523/jneurosci.22-02-00464.2002 11784791 PMC6758652

[B119] Youn Y. H. Pramparo T. Hirotsune S. Wynshaw-Boris A. (2009). Distinct dose-dependent cortical neuronal migration and neurite extension defects in Lis1 and Ndel1 mutant mice. *J. Neurosci.* 29 15520–15530. 10.1523/jneurosci.4630-09.2009 20007476 PMC2824645

[B120] Yue Y. Zong W. Li X. Li J. Zhang Y. Wu R. (2020). Long-term, in toto live imaging of cardiomyocyte behaviour during mouse ventricle chamber formation at single-cell resolution. *Nat. Cell Biol.* 22 332–340. 10.1038/s41556-020-0475-2 32123336

[B121] Zong H. Espinosa J. S. Su H. H. Muzumdar M. D. Luo L. (2005). Mosaic analysis with double markers in mice. *Cell* 121 479–492. 10.1016/j.cell.2005.02.012 15882628

